# Is outdoor-resting behaviour in malaria vectors consistent? Short report from northern Ghana

**DOI:** 10.12688/aasopenres.13317.2

**Published:** 2022-02-28

**Authors:** Majidah Hamid-Adiamoh, Davis Nwakanma, Isaac Sraku, Alfred Amambua-Ngwa, Yaw A. Afrane

**Affiliations:** 1 Disease Control and Elimination, MRC Unit The Gambia at the London School of Hygiene & Tropical Medicine, Banjul, The Gambia; 2 West African Centre for Cell Biology of Infectious Pathogens (WACCBIP), Department of Biochemistry, Cell and Molecular,, University of Ghana, Accra, Ghana; 3 Department of Medical Microbiology, University of Ghana Medical School, Accra, Ghana

**Keywords:** Mark-release-recapture, resting behaviour, outdoor resting, malaria vectors, northern Ghana.

## Abstract

**Background:** Recent studies have observed vectors resting predominantly outdoors in settings where anti-vector tools are extensively deployed. This has been attributed to selection pressure from use of control tools. This present study examined if the outdoor resting behaviour in the vector population is random or indicative of a consistent preference of one resting site over the other.

**Methods:** Mark-release-recapture experiments were conducted with outdoor-resting *Anopheles gambiae* and *An. funestus* mosquitoes collected from two villages in northern Ghana during rainy and dry seasons. Mosquitoes were marked with fluorescent dyes and released indoors. The experiments were controlled with indoor-resting mosquitoes, which were marked and released outdoors. Twelves release events were conducted for outdoor-resting mosquitoes and two for indoor mosquitoes, with ten replicates in each event. Species of all recaptured mosquitoes were identified and assessed for consistency in their resting behaviour.

**Results:** A total of 4,460 outdoor-resting mosquitoes comprising *An. gambiae sensu lato (s.l*.) (2,636, 59%) and *An. funestus* complex (1,824, 41%) were marked and released. Overall, 31 (0.7%) mosquitoes were recaptured mostly from outdoor location comprising 25 (81%) *An. gambiae s.l.* and 6 (19%) *An. funestus* complex. Only 3 (10%) of the recaptured mosquitoes were found resting indoors where they were released. The majority of the outdoor-recaptured mosquitoes were *An. arabiensis* (11, 39%), followed by *An. coluzzii* (7, 25%); whereas all indoor-recaptured mosquitoes were *An. coluzzii*. For the control experiment, 324 indoor-resting mosquitoes constituting 313 (97%) *An. gambiae s.l.* and 11 (3%) *An. funestus* complex were marked and released. However, none of these was recaptured neither indoors nor outdoors. More mosquitoes were captured and recaptured during rainy season, but this was not statistically significant (Z=0.79, P=0.21).

**Conclusions:** These results suggested the tendency for the mosquitoes to retain their outdoor-resting behaviour. Further investigations are required to ascertain if emerging preference for outdoor resting behaviour in malaria vector populations is consistent or a random occurrence.

## Introduction

Vector control with indoor residual spraying (IRS) and long-lasting insecticidal nets (LLINs) has contributed largely to reduction in malaria incidence and mortality ^1^. These tools were estimated to previously avert 10 % (IRS) and 68% (LLINs) malaria cases respectively ^2^. Both IRS and LLINs are insecticide-based, and they target vectors that feed and rest indoors ^3– 5^. Unfortunately, recent reports are indicating that the tools are inducing behavioural changes in mosquitoes, such that some vector species have shifted from indoor-resting behaviour to resting outdoors. This was as documented from studies in Ethiopia ^6^, Ghana ^7, 8^, Kenya ^9, 10^, Gabon ^11^ and Tanzania ^12, 13^. Such behavioural diversification in vector population can be detrimental to vector control ^14, 15^, which mainly target vectors resting indoors. Therefore, efforts to understand emergence of such behaviour are highly essential for the elimination agenda.

Outdoor resting behaviour in vector populations may be triggered by heavy use of insecticide-based indoor intervention in settings where control tools are widely implemented ^12, 16, 17^. Vectors in these settings avoid contact with insecticides and rest outdoors where no insecticide is used ^12, 16– 18^. This behaviour was described as behavioural avoidance ^19– 21^, where access to unprotected humans outdoors promotes outdoor feeding in vectors with consequential outdoor transmission ^12, 22^. Indeed, outdoor transmission has shown to accentuate residual transmission ^9, 23, 24^; where transmission persists despite high coverage of interventions ^25^. A recent estimate predicted that outdoor biting in vector populations could increase malaria cases by about 10.6 million (58.25%) across Africa in a year ^24^

Mark-release-recapture (MRR) studies are a standard experimental method extensively employed to investigate mosquito biology, ecology, life history and behaviour, measuring parameters including dispersal and flight distance; survival, population size and density, as well as blood feeding, host seeking and reproductive behaviour ^26– 29^. MRR experiments have been used particularly for field and laboratory studies involving mosquito species such as *Aedes* ^30, 31^, *Culex* ^32, 33^ and *Anopheles* ^26, 27, 29^; to collect data highly relevant in understanding pathogen transmission, gene flow and development and optimization of vector intervention tools ^27, 29, 34^.

Northern Ghana has documented outdoor resting in *An. gambiae sensu lato (s.l*.) population ^7, 8^ but it is not clear whether this behaviour only occurs randomly in the vector population. The aim of this present study was to examine if the preference for outdoor resting in the vector population was consistent or not. MRR experiments were conducted using field-collected adult mosquitoes of unknown age in two villages in northern Ghana, where coverage of IRS and LLINs are high ^8, 35, 36^.

## Methods

### Study sites

The MRR experiments were conducted in two villages in northern Ghana, Kpalsogu (9.33 ^0^ N, 1.02 ^0^ W) and Libga (9.35 ^0^ N, 0.51 ^0^ W) during the rainy season (July-November 2017) and dry season (December-January 2018). The region has a unimodal rainfall pattern with monthly density between 150-250 mm and a forest vegetation zone with mean daily temperature ranging between 25-30°C; and an average relative humidity between 65-75% ^37^.

This region was chosen for this study because of the previous reports of residual malaria transmission ^36, 38^ and the recent observation of outdoor resting in the vector populations ^7, 8^ in the region. Recently, malaria incidence rate of about 40% was documented in under-five children ^36, 38^ and LLINs coverage was estimated to be >70% in both villages selected for this study ^8, 36^. Kpasolgu has been under IRS implementation since 2008 ^8^. Extensive rice and tomatoes farming is practiced in both villages, occurring throughout the year as the villages are close to irrigation dams.

### Mosquito collection

Live outdoor-resting adult female *Anopheles* mosquitoes were collected from pit shelters, granaries and animal resting shelters. One granary and one animal shelter each were selected per compound. Six pit shelters were also dug, which were positioned about 5-10m outside six randomly selected compounds in each village. These compounds were selected if they were 50m apart and provided good shade for mosquitoes to hide. The mosquitoes were carefully collected using mouth and Prokopack aspirators (Model 419, John W. Hock Company) so that they remain alive. Twelve collections were done throughout the study period with six from each village. To compare the trend in behaviour of mosquitoes when resting indoors and also as a control for the experiments, indoor-resting mosquitoes were also collected from sleeping rooms in the same compounds where the pit shelters were situated and where the outdoor mosquitoes were collected from, using the same collection methods as described above. Two collections were done with both from Kpalsogu. Collection was done once weekly during the early mornings (05.00-07.00) for four months

### Mosquito processing, marking and subsequent release

Captured mosquitoes were immediately transported to the insectary for morphological identification as female *An. gambiae s.l.* and *An. funestus* complex ^39^. Following identification, indoor and outdoor mosquitoes were carefully kept in separate cages in the insectary, irrespective of their abdominal status. The mosquitoes were kept in the insectary for about 7–8 hours during which they were fed with 10% glucose until experiment time at dusk of the same day between 18:00 – 19:00.

The marking of mosquitoes was carried out 3 hours prior to release time. Batches of 15-20 mosquitoes from the same compounds were put into the same paper cup. The outdoor-collected mosquitoes, designated as ‘test’ mosquitoes, were dusted with red fluorescent dye (BioQuip Products, Inc. California, USA) while the indoor mosquitoes, serving as control, were similarly marked with green dye (BioQuip Products, Inc. California, USA). All mosquitoes were left in the cup to rest after marking, until when transported to the study sites for release.

Two random compounds were selected near the pit traps for each release event. Between 200-250 test mosquitoes per species complex were released in five sleeping rooms (indoors) in each of these compounds. The mosquitoes were released indoors to assess if they would remain indoors to rest or return outdoors where they were originally collected from. All female *An. gambiae s.l.* and *An. funestus* complex mosquitoes collected per day were released at the same time irrespective of their number.

The control mosquitoes were released outdoors; outside one of the selected compounds. Two release events were conducted for the control mosquitoes where approx. 150 specimens were released in each release event. These mosquitoes were released outdoors to ascertain if an alternate trend in the resting behaviour would be demonstrated.

### Recapture of released mosquitoes

The mosquitoes were left for 48 hours before recapture was initiated. This offered sufficient time for the mosquitoes to redistribute in their new environment and enabled us to assess if the mosquitoes opted to return to their initial collection location.

To recapture, mosquitoes were sought from sleeping rooms, animal houses, clay pots, granaries and pit traps deploying light traps, exit traps, pyrethrum spray catches as well as mouth and Prokopak aspirations. All rooms and compounds in each of the study sites were sampled to recapture the released mosquitoes. Recapturing was done for up to 7 consecutive days before the next release event.

In all, twelve (12) rounds of MRR experiments were conducted for the test mosquitoes within the study period, with six experiments performed per study site. Five of the seven experiments during the rainy season were done at Kpalsogu, whereas four out of the five experiments done in the dry season took place at Libga. Two control MRR experiments were also conducted, one each during rainy and dry season at Kpalsogu.

### Processing and identification of recaptured mosquitoes

All recaptured mosquitoes were morphologically identified as marked and unmarked. Only the marked *Anopheles* mosquitoes were selected for analyses and assessed for change in resting behaviour. All unmarked mosquitoes were only counted and recorded.

The marked recaptured mosquitoes were processed to discriminate the sibling members of *An. gambiae s.l.* and *An. funestus* complex. DNA was extracted from these mosquitoes and molecular species were genotyped using the protocols previously described ^40, 41^.

### Ethical considerations

Ethical approval was obtained from the Ghana Institutional Review Boards (IRB) of Noguchi Memorial Institute for Medical Research (NMIMR-Study #106/16-17). Village heads were also engaged in discussions where concerns on release of mosquitoes into sleeping rooms were clarified. A series of meetings with the village heads, household heads and village health workers during which the nature, benefits, and absence of risks from this study was explained. Subsequently, verbal informed consent was obtained from household heads before mosquitoes were released into their rooms. The IRB approved this type of consent taking. MRR experiments present no potential risks as mosquitoes were released in the compounds where they were initially captured and a proportion was to be recaptured. There were no children sleeping in any of the rooms where mosquitoes were released during the study period.

### Data analysis

The number of recaptured mosquitoes was counted and proportion determined. The different release events were considered as replicates. Data from both study sites were merged together as there was no significant difference in the results obtained from the different replicates. Z-test was used to compare the difference in proportion of the recaptured mosquitoes between the rainy and dry season. Stata/IC 15.0 (2017 StataCorp LP) was used for all statistical analyses.

## Results

### Recaptured mosquitoes and their species

**Figure 1.**
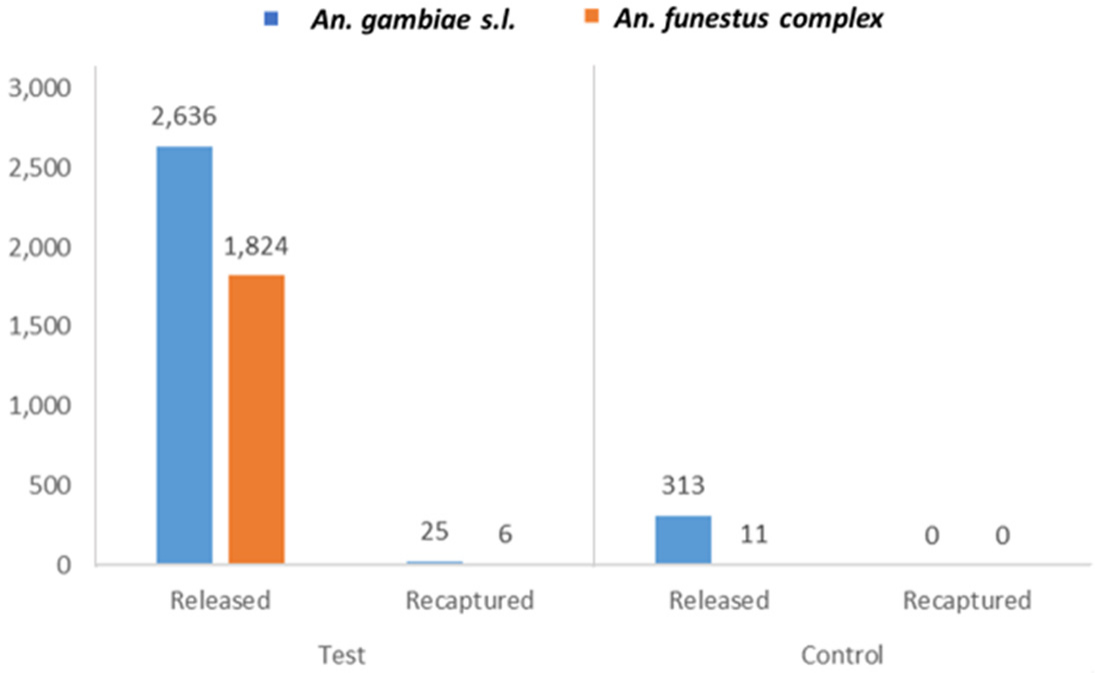
Composition of released and recaptured test and control mosquitoes. "Test" (left) are mosquitoes collected from outdoor and released indoors, whereas "Control" (right) are those mosquitoes collected from indoor and released outdoors. "Test" mosquitoes are the mosquitoes being investigated for consistency or randomness in resting behaviour. "Control" mosquitoes are the counterpart used to confirm alternate trend in the behaviour of the “test” mosquitoes.

Overall, a total of 4,460 outdoor-resting test mosquitoes were released to indoor space, while 324 indoor-resting control mosquitoes were released outdoors. The morphological identification of the released (test) mosquitoes revealed 2,636 (59%) were *An. gambiae s.l.*, while 1,824 (41%) were *An. funestus* complex ( Figure 1). The control mosquitoes comprised 313 (97%) *An. gambiae s.l.* and 11 (3%) *An. funestus* complex. The number of mosquitoes released per experiment from each of the study sites is shown in Table 1 (Test) and Table 2 (Control).

**Table 1.** Distribution of test mosquitoes released and recaptured per study site. The seven experiments conducted in Kpalsogu were mainly during rainy season and the five experiments in Libga were done mainly in dry season.

Experiment ID	Study sites	Number of mosquitoes released		Number of marked mosquitoes recaptured	
		*An. gambiae* *s.l.*	*An. funestus* *complex*	Indoor-resting	Outdoor- resting
1	Kpalsogu	227	118	0	3
2	Kpalsogu	228	178	0	4
3	Kpalsogu	221	168	1	3
4	Kpalsogu	244	223	0	3
5	Libga	221	182	0	3
6	Libga	251	173	0	0
7	Kpalsogu	224	145	1	2
8	Kpalsogu	239	181	0	2
9	Libga	206	215	1	5
10	Libga	159	136	0	0
11	Libga	223	77	0	3
12	Libga	193	28	0	0

**Table 2.** Distribution of control mosquitoes released and recaptured. The two control experiments were conducted in Kpalsogu only, one each in rainy and dry season. No mosquito was recaptured.

Experiment ID	Study sites	Number of mosquitoes released		Number of marked mosquitoes recaptured	
		*An. gambiae* *s.l.*	*An. funestus* *complex*	Indoor-resting	Outdoor-resting
1	Kpalsogu	161	9	0	0
2	Kpalsogu	152	2	0	0

Following all the 12 MRR experiments conducted, a total of 3, 950 mosquitoes were collected from outdoor location, and 141 from indoor location. Of the outdoor-caught mosquitoes, 31 (0.8%) were marked ( Table 1) and 3,919 (99.2%) were unmarked including males and non-Anophelines. Only the marked mosquitoes, which were identified to have been recaptured after being released were subsequently analysed.

Recapture of the test mosquitoes was achieved from nine (9) out of twelve release experiments. Thirty-one (31) marked test mosquitoes were recaptured from 4,460 released. This indicates a recapture rate of 0.7%. More mosquitoes (17, 55%) were recaptured from Kpalsogu during the rainy season than from Libga (14, 45%), where MRR was mostly done during the dry season. The difference in recapture rate between the study sites, as well as the rainy and dry seasons was not statistically significant (Z=0.79, P=0.21).

The breakdown of the recaptured test mosquitoes comprised 25 (81%) *An. gambiae s.l.* and 6 (19%) *An. funestus* complex ( Figure 1), from which 28 (90%) were resting outdoors, and 3 (10%) indoors. The majority of the mosquitoes recaptured outdoors were *An. arabiensis* (11, 39%), followed by *An. coluzzii* (7, 25%) and *An. funestus s.s.* (6, 21%). *An. gambiae s.s.* constituted the least outdoor-recaptured species (4, 15%) ( Table 3). The three mosquitoes recaptured indoors were all identified as *An. coluzzii*. None of the control samples that were released outdoors was recaptured from any location, indoor or outdoor.

**Table 3.** Molecular species of the recaptured test mosquitoes. These were the marked test mosquitoes only, 28 of which were recaptured outdoors and 3 indoors.

	# Recaptured	
**Mosquito species**	Indoor	Outdoor
*An. arabiensis*	0	11
*An. coluzzii*	3	7
*An. gambiae s.s.*	0	4
*An. funestus s.s.*	0	6

## Discussion

Behavioural shift from predominantly indoor to outdoor-resting is becoming widespread in vector populations in settings where anti-vector vector interventions are extensively deployed ^7, 16, 17, 42, 43^. The aim of this study was to examine if outdoor resting behaviour of the malaria vector populations described in the study settings ^7, 8^ was consistent or it was just a random occurrence. The study observed that among the test mosquitoes recaptured, the majority was collected outdoors. This suggested that these mosquitoes returned outdoors to rest after being released indoors. This pattern of mosquitoes returning outdoors was observed from nine out of the twelve experiments, despite a low recapture rate. As outdoor resting behaviour in vector population could counteract control efforts ^22, 23, 25^, research assessing the extent of this behaviour is well-timed and necessary to guide decision making in malaria control programmes.

Switch to outdoor-resting behaviour in the vectors may be as a result of avoidance behaviour from indoor interventions ^25, 44^, which were heavily used in the study sites. Most sleeping rooms had LLINs and they were also sprayed few months before the study with pirimiphos methyl, the insecticide that was being used for IRS at the time of the experiments ^8^. These mosquitoes might be avoiding contact with the insecticide indoors and chose to rest outdoors, where they may be safe, as there was no outdoor intervention in the villages. This could have also accounted for the predominant outdoor resting preference documented in the vectors in a previous study from this setting ^7^ and others all over Africa ^7, 9, 13, 23, 43^, which prompted this investigation.

The highly endophilic vectors, *An. gambiae s.s., An. coluzzii* and *An. funestus s.s.* were recaptured outdoors after being released indoors. This suggests a likely switch to outdoor resting behaviour because these vector species may be avoiding contact with insecticide indoors and exiting to rest outdoors ^45– 47^. Change from endophily (preference for indoor resting) to exophily (preference for outdoor resting) in response to control intervention is increasingly being documented in these highly endophilic vectors ^9, 13, 17, 42, 48, 49^. This could have negative impact on malaria control as it can promote outdoor and residual transmission in these settings ^12, 25, 50^.

*Anopheles arabiensis* was found to predominate the mosquito species found resting outdoors from the recaptured test mosquitoes. This may be because *An. arabiensis* is known to be highly exophilic and zoophilic (preference for animal blood meal) ^51– 53^, where it tends to stays outdoors to rest and feed on animals when there is intervention indoors ^5, 54^. This vector species has also been observed to display insecticide avoidance and early-exiting behaviour ^13, 22, 47^, which make them difficult to control ^12^

The study recorded a much lower recapture rate than most MRR studies reported. Several reasons could have accounted for this. One is that the intervention indoors could have could have killed the mosquitoes while attempting to rest post-feeding ^55, 56^. Another reason is the age of mosquitoes ^27, 29, 57^, which was unknown in this study. Regrettably, the study could not determine the age of the mosquitoes used in the study due to technical issues. Plausibly, if the mosquitoes were old, they may have died within the period of recapture. Indeed, senescence is a factor associated with reduction of mosquito daily survival in the wild ^58, 59^. Age was also previously suggested to be responsible for the low recapture rate in a similar experiment ^27^. Moreover, predators such as spiders were particularly common in the study areas, and were found inside animal houses that provide favourable environment for the mosquitoes to rest. Likewise, as some of the released mosquitoes were blood fed as well as semi-gravid and gravid, they might divert to seeking favourable oviposition spots which naturally occurs in the night. These mosquitoes may not return to rest within the same locality or might have been exposed to predators or died naturally. Other factors including emigration from the study area, climate condition, stress and negative effect of the experimental procedures could have also contributed to this low recapture success as previously documented in other MRR experiments ^27, 29, 34^.

The study also recaptured more mosquitoes during the rainy season relative to the dry season. This might be because more mosquitoes were released during this rainy season as expected. However, none of the control mosquitoes was recaptured throughout the experiments. The possible explanations could be that the mosquitoes might have died due to the residual effect of insecticides ^55, 56^ from their initial contact before collection. It may also be due to predator attack and other factors as suggested above.

The study acknowledges the limitations in the sample size and inability to determine the age of mosquitoes used in this study. Data on the abdominal status were also not collected, which could have explained the low recapture rate observed. Likewise, due to cost and logistic reasons, more MRR experiments could not be conducted equally at the study sites during each season.

## Conclusions

This study suggests that preference for outdoor resting in malaria vectors may be an emerging possibly consistent behaviour. This could have negative implication for malaria control in the study sites which currently implement only indoor interventions. There is a need for further studies to establish this observation in settings where interventions are extensively deployed. Furthermore, a probe into the genetic basis underlying this behavioural change will also be highly essential. This is important as malaria control moves to the elimination phase in sub-Saharan African countries.

## Data availability

### Underlying data

Open Science Framework: Is outdoor-resting behaviour in malaria vectors consistent? Short report from northern Ghana, https://doi.org/10.17605/OSF.IO/HFRVW ^55^.

This project contains the following underlying data:

- Raw datasheet.xlsx
- Species Identification_Raw.xlsx

Data are available under the terms of the Creative Commons Zero "No rights reserved" data waiver (CC0 1.0 Public domain dedication).

## References

[1] World Health Organization: World Malaria Report.WHO/HTM/GM, World Health,2020;238. Reference Source

[2] Bhatt S Weiss DJ Cameron E : The effect of malaria control on *Plasmodium falciparum* in Africa between 2000 and 2015. *Nature.* 2015;526(7572):207–211. 10.1038/nature15535 26375008 PMC4820050

[3] Githeko AK Service MW Mbogo CM : Resting behaviour, ecology and genetics of malaria vectors in large scale agricultural areas of Western Kenya. *Parassitologia.* 1996;38(3):481–9. 9257337

[4] Githeko AK Adungo NI Karanja DM : Some observations on the biting behavior of *Anopheles gambiae s.s.*, *Anopheles arabiensis*, and *Anopheles funestus* and their implications for malaria control. *Exp Parasitol.* 1996;82(3):306–15. 10.1006/expr.1996.0038 8631382

[5] Coluzzi M Sabatini A Petrarca V : Behavioural divergences between mosquitoes with different inversion karyotypes in polymorphic populations of the *Anopheles gambiae* complex. *Nature.* 1977;266(5605):832–3. 10.1038/266832a0 865604

[6] Kibret S Wilson GG : Increased outdoor biting tendency of *Anopheles arabiensis* and its challenge for malaria control in Central Ethiopia. *Public Health.* 2016;141:143–145. 10.1016/j.puhe.2016.09.012 27931990

[7] Hamid-Adiamoh M Amambua-Ngwa A Nwakanma D : Insecticide resistance in indoor and outdoor-resting *Anopheles gambiae* in Northern Ghana. *Malar J.* 2020;19(1):314. 10.1186/s12936-020-03388-1 32867769 PMC7460795

[8] Coleman S Dadzie SK Seyoum A : A reduction in malaria transmission intensity in Northern Ghana after 7 years of indoor residual spraying. *Malar J.* 2017;16(1):324. 10.1186/s12936-017-1971-0 28797269 PMC5553800

[9] Degefa T Yewhalaw D Zhou G : Indoor and outdoor malaria vector surveillance in western Kenya: Implications for better understanding of residual transmission. *Malar J.* 2017;16(1):443. 10.1186/s12936-017-2098-z 29110670 PMC5674686

[10] Machani MG Ochomo E Amimo F : Resting behaviour of malaria vectors in highland and lowland sites of western Kenya: Implication on malaria vector control measures. *PLoS One.* 2020;15(2):e0224718. 10.1371/journal.pone.0224718 32097407 PMC7041793

[11] Mourou JR Coffinet T Jarjaval F : Malaria transmission in Libreville: results of a one year survey. *Malar J.* 2012;11:40. 10.1186/1475-2875-11-40 22321336 PMC3310827

[12] Killeen GF Govella NJ Lwetoijera DW : Most outdoor malaria transmission by behaviourally-resistant *Anopheles arabiensis* is mediated by mosquitoes that have previously been inside houses. *Malar J.* 2016;15:225. 10.1186/s12936-016-1280-z 27093890 PMC4837512

[13] Kreppel KS Viana M Main BJ : Emergence of behavioural avoidance strategies of malaria vectors in areas of high LLIN coverage in Tanzania. *Sci Rep.* 2020;10(1):14527. 10.1038/s41598-020-71187-4 32883976 PMC7471940

[14] Sougoufara S Ottih EC Tripet F : The need for new vector control approaches targeting outdoor biting Anopheline malaria vector communities. *Parasit Vectors.* 2020;13(1):295. 10.1186/s13071-020-04170-7 32522290 PMC7285743

[15] Sougoufara S Doucouré S Backé Sembéne PM : Challenges for malaria vector control in sub-Saharan Africa: Resistance and behavioral adaptations in *Anopheles* populations. *J Vector Borne Dis.* 2017;54(1):4–15. 28352041

[16] Gatton ML Chitnis N Churcher T : The importance of mosquito behavioural adaptations to malaria control in Africa. *Evolution.* 2013;67(4):1218–30. 10.1111/evo.12063 23550770 PMC3655544

[17] Sokhna C Ndiath MO Rogier C : The changes in mosquito vector behaviour and the emerging resistance to insecticides will challenge the decline of malaria. *Clin Microbiol Infect.* 2013;19(10):902–7. 10.1111/1469-0691.12314 23910459

[18] Carrasco D Lefèvre T Moiroux N : Behavioural adaptations of mosquito vectors to insecticide control. *Curr Opin Insect Sci.* 2019;34:48–54. 10.1016/j.cois.2019.03.005 31247417

[19] Durnez L Mao S Denis L : Outdoor malaria transmission in forested villages of Cambodia. *Malar J.* 2013;12:329. 10.1186/1475-2875-12-329 24044424 PMC3848552

[20] Gryseels C Durnez L Gerrets R : Re-imagining malaria: heterogeneity of human and mosquito behaviour in relation to residual malaria transmission in Cambodia. *Malar J.* 2015;14:165. 10.1186/s12936-015-0689-0 25908498 PMC4408599

[21] Sande S Zimba M Chinwada P : Insights Into Resting Behavior of Malaria Vector Mosquitoes in Mutare and Mutasa Districts of Manicaland Province, Zimbabwe. *J Med Entomol.* 2016;53(4):866–72. 10.1093/jme/tjw044 27134207

[22] Killeen GF Marshall JM Kiware SS : Measuring, manipulating and exploiting behaviours of adult mosquitoes to optimise malaria vector control impact. *BMJ Glob Health.* 2017;2(2):e000212. 10.1136/bmjgh-2016-000212 28589023 PMC5444085

[23] Russell TL Govella NJ Azizi S : Increased proportions of outdoor feeding among residual malaria vector populations following increased use of insecticide-treated nets in rural Tanzania. *Malar J.* 2011;10:80. 10.1186/1475-2875-10-80 21477321 PMC3084176

[24] Sherrard-Smith E Skarp JE Beale AD : Mosquito feeding behavior and how it influences residual malaria transmission across Africa. *Proc Natl Acad Sci U S A.* 2019;116(30):15086–15095. 10.1073/pnas.1820646116 31285346 PMC6660788

[25] Killeen GF : Characterizing, controlling and eliminating residual malaria transmission. *Malar J.* 2014;13:330. 10.1186/1475-2875-13-330 25149656 PMC4159526

[26] Saddler A Kreppel KS Chitnis N : The development and evaluation of a self-marking unit to estimate malaria vector survival and dispersal distance. *Malar J.* 2019;18(1):441. 10.1186/s12936-019-3077-3 31870365 PMC6929409

[27] Epopa PS Millogo AA Collins CM : The use of sequential mark-release-recapture experiments to estimate population size, survival and dispersal of male mosquitoes of the *Anopheles gambiae* complex in Bana, a west African humid savannah village. *Parasit Vectors.* 2017;10(1):376. 10.1186/s13071-017-2310-6 28784147 PMC5547516

[28] Service MW : Mosquito ecology: Field sampling methods.Second edition.1993. 10.1007/978-94-011-1868-2

[29] Guerra CA Reiner RC Jr Perkins TA : A global assembly of adult female mosquito mark-release-recapture data to inform the control of mosquito-borne pathogens. *Parasit Vectors.* 2014;7:276. 10.1186/1756-3305-7-276 24946878 PMC4067626

[30] Vavassori L Saddler A Müller P : Active dispersal of *Aedes albopictus*: A mark-release-recapture study using self-marking units. *Parasit Vectors.* 2019;12(1):583. 10.1186/s13071-019-3837-5 31831040 PMC6909613

[31] Russell RC Webb CE Williams CR : Mark-release-recapture study to measure dispersal of the mosquito *Aedes aegypti* in Cairns, Queensland, Australia. *Med Vet Entomol.* 2005;19(4):451–7. 10.1111/j.1365-2915.2005.00589.x 16336310

[32] Niebylski ML Meek CL : A self-marking device for emergent adult mosquitoes. *J Am Mosq Control Assoc.* 1989;5(1):86–90. 2565370

[33] Ciota AT Drummond CL Ruby MA : Dispersal of *Culex* mosquitoes (Diptera: Culicidae) from a wastewater treatment facility. *J Med Entomol.* 2012;49(1):35–42. 10.1603/me11077 22308769 PMC3278816

[34] Midega JT Mbogo CM Mwambi H : Estimating dispersal and survival of *anopheles gambiae* and *anopheles funestus* along the Kenyan coast by using mark-release-recapture methods. *J Med Entomol.* 2007;44(6):923–9. 10.1603/0022-2585(2007)44[923:edasoa]2.0.co;2 18047189 PMC2705338

[35] Monroe A Asamoah O Lam Y : Outdoor-sleeping and other night-time activities in northern Ghana: Implications for residual transmission and malaria prevention. *Malar J.* 2015;14(1):35. 10.1186/s12936-015-0543-4 25627277 PMC4320825

[36] Abuaku B Ahorlu C Psychas P : Impact of indoor residual spraying on malaria parasitaemia in the Bunkpurugu-Yunyoo District in northern Ghana. *Parasit Vectors.* 2018;11(1):555. 10.1186/s13071-018-3130-z 30352613 PMC6199755

[37] McSweeney C New M Lizcano G : The UNDP climate change country profiles. *Bull Am Meteorol Soc.* 2010;91(2):157–166. 10.1175/2009BAMS2826.1

[38] Millar J Psychas P Abuaku B : Detecting local risk factors for residual malaria in northern Ghana using Bayesian model averaging. *Malar J.* 2018;17(1):343. 10.1186/s12936-018-2491-2 30268127 PMC6162921

[39] Gillies MT Coetzee M : A Supplement to the Anophelinae of the South of the Sahara (Afrotropical Region). *Publications of the South African Institute for Medical Research.* 1987;55:1–143. Reference Source

[40] Koekemoer LL Kamau L Hunt RH : A cocktail polymerase chain reaction assay to identify members of the Anopheles funestus (Diptera: Culicidae) group. *Am J Trop Med Hyg.* 2002;66(6):804–11. 10.4269/ajtmh.2002.66.804 12224596

[41] Fanello C Santolamazza F della Torre A : Simultaneous identification of species and molecular forms of the *Anopheles gambiae* complex by PCR-RFLP. *Med Vet Entomol.* 2002;16(4):461–4. 10.1046/j.1365-2915.2002.00393.x 12510902

[42] Reddy MR Overgaard HJ Abaga S : Outdoor host seeking behaviour of Anopheles gambiae mosquitoes following initiation of malaria vector control on Bioko Island, Equatorial Guinea. *Malar J.* 2011;10:184. 10.1186/1475-2875-10-184 21736750 PMC3146901

[43] Meyers JI Pathikonda S Popkin-Hall ZR : Increasing outdoor host-seeking in *Anopheles gambiae* over 6 years of vector control on Bioko Island. *Malar J.* 2016;15(1):239. 10.1186/s12936-016-1286-6 27113244 PMC4845310

[44] Durnez L Coosemans M : Residual transmission of malaria: an old issue for new approaches.BT - *Anopheles mosquitoes – New insights into malaria vectors*.2013. 10.5772/55925

[45] Shcherbacheva A Haario H Killeen GF : Modeling host-seeking behavior of African malaria vector mosquitoes in the presence of long-lasting insecticidal nets. *Math Biosci.* 2018;295:36–47. 10.1016/j.mbs.2017.10.005 29031707

[46] Killeen GF Kiware SS Okumu FO : Going beyond personal protection against mosquito bites to eliminate malaria transmission: population suppression of malaria vectors that exploit both human and animal blood. *BMJ Glob Health.* 2017;2(2):e000198. 10.1136/bmjgh-2016-000198 28589015 PMC5444054

[47] Govella NJ Chaki PP Killeen GF : Entomological surveillance of behavioural resilience and resistance in residual malaria vector populations. *Malar J.* 2013;12:124. 10.1186/1475-2875-12-124 23577656 PMC3637503

[48] Sougoufara S Sokhna C Diagne N : The implementation of long-lasting insecticidal bed nets has differential effects on the genetic structure of the African malaria vectors in the *Anopheles gambiae* complex in Dielmo, Senegal. *Malar J.* 2017;16(1):337. 10.1186/s12936-017-1992-8 28810861 PMC5558778

[49] Sanou A Nelli L Guelbéogo WM : Insecticide resistance and behavioural adaptation as a response to long-lasting insecticidal net deployment in malaria vectors in the Cascades region of Burkina Faso. *Sci Rep.* 2021;11(1):17569. 10.1038/s41598-021-96759-w 34475470 PMC8413378

[50] Bamou R Mbakop LR Kopya E : Changes in malaria vector bionomics and transmission patterns in the equatorial forest region of Cameroon between 2000 and 2017. *Parasit Vectors.* 2018;11(1):464. 10.1186/s13071-018-3049-4 30103825 PMC6090627

[51] Massebo F Balkew M Gebre-Michael T : Zoophagic behaviour of anopheline mosquitoes in southwest Ethiopia: Opportunity for malaria vector control. *Parasit Vectors.* 2015;8(1):645. 10.1186/s13071-015-1264-9 26684464 PMC4684615

[52] Main BJ Lee Y Ferguson HM : The Genetic Basis of Host Preference and Resting Behavior in the Major African Malaria Vector, *Anopheles arabiensis.* *PLoS Genet.* 2016;12(9):e1006303. 10.1371/journal.pgen.1006303 27631375 PMC5025075

[53] Bryan JH Petrarca V Di Deco MA : Adult behaviour of members of the Anopheles gambiae complex in the Gambia with special reference to An. melas and its chromosomal variants. *Parassitologia.* 1987;29(2–3):221–49. 3508262

[54] Coluzzi M Sabatini A Petrarca V : Chromosomal differentiation and adaptation to human environments in the *anopheles gambiae* complex. *Trans R Soc Trop Med Hyg.* 1979;73(5):483–97. 10.1016/0035-9203(79)90036-1 394408

[55] Brady OJ Godfray HC Tatem AJ : Adult vector control, mosquito ecology and malaria transmission. *Int Health.* 2015;7(2):121–9. 10.1093/inthealth/ihv010 25733562 PMC4357799

[56] Killeen GF Seyoum A Sikaala C : Eliminating malaria vectors. *Parasit Vectors.* 2013;6:172. 10.1186/1756-3305-6-172 23758937 PMC3685528

[57] Cho SH Lee HW Shin EH : A mark-release-recapture experiment with *Anopheles sinensis* in the northern part of Gyeonggi-do, Korea. *Korean J Parasitol.* 2002;40(3):139–48. 10.3347/kjp.2002.40.3.139 12325443 PMC2721040

[58] Clements AN Paterson GD : The Analysis of Mortality and Survival Rates in Wild Populations of Mosquitoes. *J Appl Ecol.* 1981;18(2):373–399. 10.2307/2402401

[59] Styer LM Carey JR Wang JL : Mosquitoes do senesce: Departure from the paradigm of constant mortality. *Am J Trop Med Hyg.* 2007;76(1):111–7. 17255238 PMC2408870

