# Supplementary material for: Is outdoor-resting behaviour in malaria vectors consistent? Short report from northern Ghana
Source: AAS Open Res. 2022 Feb 28;4:53. Originally published 2021 Nov 16. [Version 2] doi: 10.12688/aasopenres.13317.2 (PMC11347917; doi:10.12688/aasopenres.13317.2)
Supplement: Peer review history

# Reviewer report: Reviewer response for version 2

**DOI:** 10.21956/aasopenres.14507.r31553

**Published:** 2024-08-26

**Authors:** Silke Fuchs

**Affiliations:** 1 Imperial College London, London, England, UK

In this report, the authors investigated the outdoor resting behaviour of *Anopheles gambiae* s.l. and *Anopheles coluzzii* s.l. mosquitoes in 2 villages in northern Ghana during the rainy and dry season. The main aim was to determine whether mosquitoes preferred to rest outdoors over indoors. This shift has already been observed in other studies and geographical locations. 

 In this particular study, the authors collected mosquitoes outdoors and investigated whether they would return to this setting after being released indoors. 

 Overall, the study was well-presented and comprised a lot of work.

 However, some results indicated limitations of the study which were not discussed enough (e.g. failure of the control, no distinction between gravid and non-gravid females, no species identification/numbers at the start of the experiment). 

 More specific comments and questions for each section include:

**Abstract:**

Method: It should say mosquitoes of the *An. gambiae* complex and *A. funestus *complex so that the text aligns with the results section.

Results: The numbers do not add up. It states 31 were collected of which 25 belonged to the *An. gambiae* complex and 6 to the *Anopheles coluzzii* complex. However, only 11 *An. arabiensis*, 7 *An. coluzzii *(found outdoors) and 3 *An. coluzzii* (found indoors) are specified in the text (total= 22 not 25). It is also not quite clear if any *An. funestus* complex mosquitoes were found indoors or outdoors.

 Since no mosquitoes were recaptured in the control, it should be stated what this means for the interpretation of the experiment. Why were the release numbers for the control 14x below the number of the indoor-released mosquitoes?

 If it is not statistically significant, then the text should not say that more were recaptured. The authors could just provide the numbers collected in the rainy versus dry season.

Conclusion: Unfortunately, it is not clear from the abstract how many houses were examined or how big the outdoor collection area was. The conclusion can state that mosquitoes released indoors rest outdoors, but the conclusion cannot imply that certain mosquitoes rest only indoors and others only rest outdoors.

**Main text**:

Methods: Mosquito processing, marking and subsequent release:

 What do the authors mean by abdominal status (whether they are males or females or whether they were gravid/ looking for a site to lay eggs?)?

 How do you know if the mosquitoes outside were not looking for oviposition sites (so had already had their bloodmeal) and the ones inside were looking for a bloodmeal?

Recapture of released mosquitoes:

 What was the distance of those recapture sites from the release site? How did you choose the 48-hour recapturing period? Was that based on previous MRRs?

 Assuming the released mosquitoes had enough time to feed within the 48 hours inside the rooms, why would they stay indoors after they have fed and rested (considering that females lay eggs ~48 hours after blood meal)?

 Were there any bed nets in use at the time they were released in those houses? If not, how would the absence of the insecticide promote their outdoor behaviour?

Processing and identification of recaptured mosquitoes

 What was the purpose of counting and recording the unmarked mosquitoes?

**Results:**

 The scale/ figure style of Figure 1 should be reconsidered so that the *An. funestus* numbers are shown in the bars.

 Is the recapture rate comparable to other MRR studies or could there be improvements/ observations that you made which could be useful for future experiments?

 Do you have more specific information on where most of the outdoor recaptured mosquitoes were found (e.g. distance from the release site, what kind of shelter)?

 Unfortunately, the species composition at the start of the experiment is not known, so that it is not possible to make conclusions as to which species is more likely to be found outdoors.

 After you recaptured no mosquitoes after the first control experiment, what changes did you make to improve your chances for detection? Why was the number of indoor collected mosquitoes so much lower than the outdoor collected ones? Is it because most mosquitoes rest outdoors? If yes, would this not indicate already a preference for outdoor resting sites rather than a random distribution?

**Discussion:**

 Were the recaptured mosquitoes able to have a blood meal indoors?

 How do you determine if the mosquito is in resting behaviour in and outdoors (did you choose specific collection sites known to be resting sites or was it the time after bloodmeal)? Why would the mosquito stay indoors more than 1 day after a potential blood meal if it has to look for a breeding site?

 Again, I do not think that a statement about a preference by a particular species can be made as the species composition of the marked mosquitoes was not determined at the beginning of the experiment. Also, the recaptured numbers are very low to make that conclusion.

 Why would the mosquitoes be older in this study than most MRRs for which higher recapture rates have been reported?

 It should have been reported in the results section how many mosquitoes were gravid as they would be expected to display a very different behaviour over those 48 hours after the release indoors. Why were gravid females included in this experiment?

 Oviposition spots are not just preferred at night but also outdoors.

 In terms of the low recapture rate, did you consider other factors such as distance from the release site, the choice of collection sites, and time after the release? Further, as acknowledged at the end of the discussion, the suitable release numbers could have been also a contributing factor (e.g. the control group was 14x lower than your tested group and no mosquitoes were recaptured).

 Unfortunately, the authors did not discuss what they would change in future experiments (more than just increasing the number of MRRs) nor what impact the failure of the control had on the interpretation of the experiment.

 This could be useful for future MRRs in this field.

Is the work clearly and accurately presented and does it cite the current literature?

Yes

If applicable, is the statistical analysis and its interpretation appropriate?

Partly

Are all the source data underlying the results available to ensure full reproducibility?

Yes

Is the study design appropriate and is the work technically sound?

Partly

Are the conclusions drawn adequately supported by the results?

Partly

Are sufficient details of methods and analysis provided to allow replication by others?

Partly

Reviewer Expertise:

Medical Entomology and Insect Molecular Biology

I confirm that I have read this submission and believe that I have an appropriate level of expertise to confirm that it is of an acceptable scientific standard, however I have significant reservations, as outlined above.

# Reviewer report: Reviewer response for version 2

**DOI:** 10.21956/aasopenres.14507.r29154

**Published:** 2022-03-01

**Authors:** Ibrahima Dia

**Affiliations:** 1 Medical Zoology Pole, Pasteur Institute of Dakar, Dakar, Senegal

The authors have addressed all the concerns raised.

Is the work clearly and accurately presented and does it cite the current literature?

Partly

If applicable, is the statistical analysis and its interpretation appropriate?

Not applicable

Are all the source data underlying the results available to ensure full reproducibility?

Partly

Is the study design appropriate and is the work technically sound?

Partly

Are the conclusions drawn adequately supported by the results?

Yes

Are sufficient details of methods and analysis provided to allow replication by others?

Partly

Reviewer Expertise:

Medical Entomology

I confirm that I have read this submission and believe that I have an appropriate level of expertise to confirm that it is of an acceptable scientific standard.

# Reviewer report: Reviewer response for version 1

**DOI:** 10.21956/aasopenres.14444.r29122

**Published:** 2022-02-11

**Authors:** Ibrahima Dia

**Affiliations:** 1 Medical Zoology Pole, Pasteur Institute of Dakar, Dakar, Senegal

The paper by Hamid-Adiamoh *et al.* deals with the problem of outdoor-resting behaviour of malaria vectors as observed in several contexts in Africa following the implementation of control strategies. In fact, even if this phenomenon is confirmed in many places in tropical Africa, including in northern Ghana, there is no evidence of its consistency or if it is a phenomenon specific to populations usually found outside human dwellings. This study was carried out in two villages in northern Ghana during the rainy season and dry season. It consisted of releasing inside the human rooms mosquitoes collected resting outside and endophilic mosquitoes outside. The major results showed a tendency for mosquitoes to retain their exophilic behavior.

 The paper is well presented and the results seem conclusive but it has some limitations:

- The two villages selected do not have the same status concerning the control measures implemented (IRS in the village of Kpasolgu since 2008), a presentation of the results by village is necessary to understand their possible effect on the exophilic behavior.
- In some parts, the authors should highlight the outdoor-resting behavior rather than residual transmission since no data on the mosquito aggressiveness to humans has been presented knowing that it is this fraction which is studied for the estimation of transmission. In addition, there is often no definite link between the resting place and the biting site, as mosquitoes can have different resting and biting behaviors.
- The lack of data on the physiological status of mosquitoes does not allow a better appreciation of the outdoor-resting behavior. Indeed, resting mosquitoes are often those that have already taken their blood meal and are looking for an appropriate site for blood digestion. For example, if exophagous mosquitoes are collected outdoors before and then released indoors, they will tend to return to their preferred biting site and vice versa. As data on the physiological state of mosquitoes are lacking, this should be discussed.
- If previous data on the vectors are available, it would be interesting to present them to allow a better understanding of vector behavior changes, especially in the village of Kpasolgu where the IRS have been carried out since 2008.

Specific remarks:

- Page 3, column 1, “Introduction section”, line 10, replace “Libreville” by Gabon to homogenize with the other countries.
- Page 3, column 1, “Introduction section”, last paragraph of the introduction section. Even if the age is unknown, please provide the physiological age if known.
- Page 3, column 1, “Methods section”, line 5, replace “density” by “amount”.
- Page 3, column 2, “Mosquito processing, marking and subsequent release”:
  In this part, we understand that the mosquitoes collected without determining their physiological age, have digested the bloodmeal they have taken and are looking for a suitable breeding site to lay their eggs (case of gravid mosquitoes) or for a host (case of unfed mosquitoes) to take a blood meal. If these data are not available, this issue should be discussed as a limitation of the study.
- Page 4, column 1, paragraph 3, line 3, remove “Most of” and give the exact number of experiments carried out in each of the two villages.
- Page 4, column 2, “Discussion section”, line 3, remove the word “vector”.
- Page 5, column 2, second paragraph, if you have existing data on vector behaviour, please provide them to better understand the switch to outdoor resting behaviour.
- Page 5, column 2, last line, the words “could have” were repeated.
- Page 5, column 1, first paragraph, the authors talk about the physiological status, while no data is given in the paper. If these data are available, they should be presented in the paper, to support the idea raised in this part.

Is the work clearly and accurately presented and does it cite the current literature?

Partly

If applicable, is the statistical analysis and its interpretation appropriate?

Not applicable

Are all the source data underlying the results available to ensure full reproducibility?

Partly

Is the study design appropriate and is the work technically sound?

Partly

Are the conclusions drawn adequately supported by the results?

Yes

Are sufficient details of methods and analysis provided to allow replication by others?

Partly

Reviewer Expertise:

Medical Entomology

I confirm that I have read this submission and believe that I have an appropriate level of expertise to confirm that it is of an acceptable scientific standard, however I have significant reservations, as outlined above.

# Response

**Published:** 2022-02-17

**Authors:** Majidah Hamid-Adiamoh

**Affiliations:** MRC Unit The Gambia at the London School of Hygiene & Tropical Medicine, The Gambia

The authors are very grateful to the reviewer for his suggestions and critiques that have improved this manuscript. Please kindly see our responses to each of the comments below.

**General**

 The paper by Hamid-Adiamoh et al. deals with the problem of outdoor-resting behaviour of malaria vectors as observed in several contexts in Africa following the implementation of control strategies. In fact, even if this phenomenon is confirmed in many places in tropical Africa, including in northern Ghana, there is no evidence of its consistency or if it is a phenomenon specific to populations usually found outside human dwellings. This study was carried out in two villages in northern Ghana during the rainy season and dry season. It consisted of releasing inside the human rooms mosquitoes collected resting outside and endophilic mosquitoes outside. The major results showed a tendency for mosquitoes to retain their exophilic behavior.

 The paper is well presented and the results seem conclusive but it has some limitations:

**Comments**: The two villages selected do not have the same status concerning the control measures implemented (IRS in the village of Kpasolgu since 2008), a presentation of the results by village is necessary to understand their possible effect on the exophilic behavior.

**Response: **Two new tables have been generated to present results by study sites.

**Comments**: In some parts, the authors should highlight the outdoor-resting behavior rather than residual transmission since no data on the mosquito aggressiveness to humans has been presented knowing that it is this fraction which is studied for the estimation of transmission. In addition, there is often no definite link between the resting place and the biting site, as mosquitoes can have different resting and biting behaviors.

**Response: **We have addressed this observation. Outdoor resting has been focused on throughout the manuscript. Residual transmission was only inferred as a consequence of outdoor feeding.

**Comment: **The lack of data on the physiological status of mosquitoes does not allow a better appreciation of the outdoor-resting behavior. Indeed, resting mosquitoes are often those that have already taken their blood meal and are looking for an appropriate site for blood digestion. For example, if exophagous mosquitoes are collected outdoors before and then released indoors, they will tend to return to their preferred biting site and vice versa. As data on the physiological state of mosquitoes are lacking, this should be discussed.

**Response: **We agree with the reviewer on this comment. We have included the limitation in determining the physiological status of the mosquitoes in this study, despite efforts to do so.

**Comment: **If previous data on the vectors are available, it would be interesting to present them to allow a better understanding of vector behavior changes, especially in the village of Kpasolgu where the IRS have been carried out since 2008.

**Response: **Unfortunately, this is not available. Only the high exophilic behaviour of mosquito population known to be highly more endophilic are available and presented in the text.

**Specific remarks**

**Comment**: Page 3, column 1, “Introduction section”, line 10, replace “Libreville” by Gabon to homogenize with the other countries.

**Response**: We have replaced Libreville with Gabon as corrected.

**Comment**: Page 3, column 1, “Introduction section”, last paragraph of the introduction section. Even if the age is unknown, please provide the physiological age if known.

**Response: **This suggestion has been incorporated. We now identify the mosquitoes released as adults.

**Comment**: Page 3, column 1, “Methods section”, line 5, replace “density” by “amount”.

**Response**: We have replaced density with amount as corrected.

**Comment**: Page 3, column 2, “Mosquito processing, marking and subsequent release”:

 In this part, we understand that the mosquitoes collected without determining their physiological age, have digested the bloodmeal they have taken and are looking for a suitable breeding site to lay their eggs (case of gravid mosquitoes) or for a host (case of unfed mosquitoes) to take a blood meal. If these data are not available, this issue should be discussed as a limitation of the study.

**Response**: This has been discussed as a limitation.

**Comment**: Page 4, column 1, paragraph 3, line 3, remove “Most of” and give the exact number of experiments carried out in each of the two villages.

**Response**: The exact numbers are now provided as suggested.

**Comment**: Page 4, column 2, “Discussion section”, line 3, remove the word “vector”.

**Response**: Vector is now removed.

**Comment**: Page 5, column 2, second paragraph, if you have existing data on vector behaviour, please provide them to better understand the switch to outdoor resting behaviour.

**Response**: The existing data we have is the predominant outdoor resting in the vector population in the same study sites and this has been cited.

**Comment**: Page 5, column 2, last line, the words “could have” were repeated.

**Response**: The second 'could' is now removed.

**Comment**: Page 5, column 1, first paragraph, the authors talk about the physiological status, while no data is given in the paper. If these data are available, they should be presented in the paper, to support the idea raised in this part.

**Response:** This data were not recorded as the study decided to release all mosquitoes irrespective of their abdominal status because of the small number collected. However, the study did observe that some of the mosquitoes were blood fed, half-gravid and gravid.

# Reviewer report: Reviewer response for version 1

**DOI:** 10.21956/aasopenres.14444.r29099

**Published:** 2022-02-10

**Authors:** Collins Kalwale Mweresa

**Affiliations:** 1 Jaramogi Oginga Odinga University of Science and Technology, Bondo, Kenya

**In general:** The final version of the revised manuscript should be proof-read and improved by a native English speaker to settle grammatical issues and provide concise and precise statements.

**Title:**

- Needs to be revised so that it can capture the study accurately. This was an experimental study.

**Background**:

- Sentence 1 and 2 should be revised to enhance logical flow.

                        

**Methods:**

- Specify the numbers of mosquitoes’ releases and numbers of replicates involved. Why didn’t you release equal numbers of *Anopheles gambiae* and *An. funestus?*

**Introduction:**

- Provide published evidence/data on the contribution of outdoor transmission to changes in malaria prevalence. Quantify the magnitude of malaria reduction due to IRS and LLLIN. Sentence two should be revised. Collect more literature on causes and mechanisms of mosquito resistance to insecticides, what was the aim and specific objectives of this study? This needs to be captured in the last paragraph.

**Methods:**

- Why was this particular study area preferred? Insert period by months in brackets for each season, what agricultural activities are supported by irrigation in the study area?
- Specify whether indoor and outdoor mosquito collections were done in the same site/compound, simultaneously, involved equal numbers of sites/compounds and state the time intervals over which the experiments were conducted.
- Specify the time interval between marking and release of the mosquitoes?
- How many mosquitoes of each species were released daily and at what frequency? How come that equal numbers of mosquito species were not released during the study? How did you deal with the variation associated with the release of unequal numbers of mosquitoes per species?
- Thus, accurate study and experimental design should be provided. How come that mosquitoes of the same age/colony were not used in this study?

**Ethical review:**

- Why were children allowed to sleep in the rooms and why was this not mentioned in the section above? And what was the inclusion and exclusion criteria used to select children for this activity? Did any of the children fall sick during the study? If so, how were they handled?
- Do not begin sentences with abbreviations e.g. MRRR or numerical values. Instead write in full/words if used at the start of the sentence. How did you confirm whether the captured and released mosquitoes were not infective?

**Results:**

- Sentence three is incomplete and needs to be revised, rephrase the legend for figure one.
- Description of test and control mosquitoes should be done accurately and transferred from the results to the methods section. Sentence six should be revised. What was the proportions of *An. gambiae s.*s and *An. Arabiensis*? And that *An. gambiae s.s* is not a species but sub-species. Table 1 needs to be revised by changing from molecular species to molecular identification of captured (...), the information provided in the same table is equally weak and should be deleted.
- Generally, the results provided in this section are not sufficient enough to make a valid and reliable conclusion.

**Discussion:**

- This section needs to be strengthened. What was the time interval between insecticide application and the onset of experiments? Account for such time interval. What new/novel information has this study generated towards malaria elimination and eradication? What next?

**Reference**:

- Ensure that all references are in conformity with the format recommended by this particular journal.

**Conclusion: **

- Suggestions made hereby should be incorporated and entire document proof-read by a native English speaker prior to indexing of this write-up.

Is the work clearly and accurately presented and does it cite the current literature?

Partly

If applicable, is the statistical analysis and its interpretation appropriate?

Partly

Are all the source data underlying the results available to ensure full reproducibility?

Partly

Is the study design appropriate and is the work technically sound?

Partly

Are the conclusions drawn adequately supported by the results?

Yes

Are sufficient details of methods and analysis provided to allow replication by others?

Partly

Reviewer Expertise:

Medical Entomology

I confirm that I have read this submission and believe that I have an appropriate level of expertise to confirm that it is of an acceptable scientific standard, however I have significant reservations, as outlined above.

# Response

**Published:** 2022-02-17

**Authors:** Majidah Hamid-Adiamoh

**Affiliations:** MRC Unit The Gambia at the London School of Hygiene & Tropical Medicine, The Gambia

The authors are very grateful to the reviewer for his suggestions and critiques that have improved this manuscripts. Please kindly see our responses to each of the comments below.

**In general**

**Comment: **The final version of the revised manuscript should be proof-read and improved by a native English speaker to settle grammatical issues and provide concise and precise statements.

**Response:** We hope the manuscript now reads well with the changes incorporated.

**Title**

**Comment:**Needs to be revised so that it can capture the study accurately. This was an experimental study.

**Response:** The title has been revised and it now reads: “Is outdoor-resting behaviour in malaria vectors consistent? Short experimental report from northern Ghana”.

**Background**

**Comment:**Sentence 1 and 2 should be revised to enhance logical flow.

**Response:** We have revised these sentences as suggested.

                    

**Methods**

**Comment: **Specify the numbers of mosquitoes’ releases and numbers of replicates involved.

**Response**: We have indicated the numbers of mosquitoes and replicates appropriately.

**Comment:** Why didn’t you release equal numbers of Anopheles gambiae and An. funestus?

**Response**: We released all the female mosquitoes collected each. We did not control the number to be released, regardless of the species. We have also discussed this as a limitation.

**Introduction**

**Comment**: Provide published evidence/data on the contribution of outdoor transmission to changes in malaria prevalence. 

**Response: **These have been provided.

**Comment**: Quantify the magnitude of malaria reduction due to IRS and LLLIN.

**Response: **These have been provided.

**Comment** : Sentence two should be revised.

**Response: **This sentence has been revised.

**Comment**: Collect more literature on causes and mechanisms of mosquito resistance to insecticides,

**Response: **We have included literatures on behavioural resistance to insecticide.

**Comment**: What was the aim and specific objectives of this study? This needs to be captured in the last paragraph.

**Response: **The aim of the study is now clearly indicated in the text.

**Methods**

**Comment**: Why was this particular study area preferred?

**Response: **We have included the reasons for the choice of the study area, which were because of the observation of predominant outdoor resting previously documented in the vectors; as well as the area being under coverage of anti-vector interventions.

**Comment**: Insert period by months in brackets for each season

**Response: **This is done accordingly.

**Comment**: What agricultural activities are supported by irrigation in the study area?

**Response: **This has been indicated as rice and tomatoes

**Comment**: Specify whether indoor and outdoor mosquito collections were done in the same site/compound, simultaneously, involved equal numbers of sites/compounds and state the time intervals over which the experiments were conducted.

**Response: **Yes, this was done simultaneously and it has been indicated. Other suggestions have also been incorporated.

**Comment**: Specify the time interval between marking and release of the mosquitoes?

**Response: **This is already indicated in the manuscript to be within 3 hours.

**Comment**: How many mosquitoes of each species were released daily and at what frequency?

**Response:** This information is already provided in the text. Again, in this revision, we have included two tables to indicate the number released and recaptured by species by study site

**Comments**: How come that equal numbers of mosquito species were not released during the study? How did you deal with the variation associated with the release of unequal numbers of mosquitoes per species? Thus, accurate study and experimental design should be provided. How come that mosquitoes of the same age/colony were not used in this study?

**Response: **We released all the female mosquitoes collected each. We did not control the number to be released, regardless of the species. We were unable to due to technical error and failed alternative arrangements. We have also discussed these as a limitation.

**Ethical review**

**Comments**: Why were children allowed to sleep in the rooms and why was this not mentioned in the section above? And what was the inclusion and exclusion criteria used to select children for this activity? Did any of the children fall sick during the study? If so,how were they handled?

**Response: ** The study did not release mosquitoes in rooms where children were sleeping. There was no children in any of the rooms used in the study.

**Comments**: Do not begin sentences with abbreviations e.g. MRRR or numerical values. Instead write in full/words if used at the start of the sentence.

**Response: **This has been duly corrected.

**Comments**: How did you confirm whether the captured and released mosquitoes were not infective?

**Response: **This was not done.

**Results**

**Comments**: Sentence three is incomplete and needs to be revised, rephrase the legend for figure one.

**Response** : This sentence has been revised.

**Comments**: Description of test and control mosquitoes should be done accurately and transferred from the results to the methods section.

**Response**: This is already done at the methods section.

**Comments:** Sentence six should be revised.

**Response**: This sentence has been revised.

**Comments:** What was the proportions of An. gambiae s.s and An. Arabiensis?

**Response** : Details of percentage of *An. gambiae s.s*. and *An. arabiensis* are also already in the manuscript. This can be seen in the text below and specifically highlighted:

 “The breakdown of the recaptured test mosquitoes comprised 25 (81%) *An. gambiae* *s.l.* and 6 (19%) *An. funestus *complex (Figure 1), from which 28 (90%) were resting outdoors, and 3 (10%) indoors. The majority of the mosquitoes recaptured outdoors were *An. arabiensis* (11, 39%), followed by *An. coluzzii* (7, 25%) and *An.* *funestus s.s.* (6, 21%). *An. gambiae s.s. *constituted the least outdoor-recaptured species (4, 15%) (Table 3)”.

**Comments:** And that An. gambiae s.s is not a species but sub-species.

**Response**: We will like to confirm that *An. gambiae s.s.* is a species not a sub-species.

**Comments: **Table 1 needs to be revised by changing from molecular species to molecular identification of captured (...), the information provided in the same table is equally weak and should be deleted.

**Response**: Authors think that molecular identification is more relevant under a method than result. We will like to retain the Table 1 as it currently is indicating the species of the mosquitoes recaptured.

**Comments**: Generally, the results provided in this section are not sufficient enough to make a valid and reliable conclusion.

**Response: **The authors appreciate the reviewer’s comment. We believe the data we present here adequately support our conclusion. We do hope that the changes we have incorporated have improved the manuscript.

**Discussion**

**Comments** This section needs to be strengthened. What was the time interval between insecticide application and the onset of experiments? Account for such time interval.

**Response** : This time interval is now indicated in the text.

**Comments**: What new/novel information has this study generated towards malaria elimination and eradication? What next?

**Response**: This study acknowledges that the findings indicate a possible consistent outdoor-resting behaviour in a setting with high indoor intervention only. The study thus recommends a parallel outdoor intervention to be implemented in such settings to complement the existing tools. Further investigations are also encouraged to establish the findings.

**Reference**

**Comments**: Ensure that all references are in conformity with the format recommended by this particular journal.

**Response: **These have been done appropriately.

**Conclusion**

**Comments**: Suggestions made hereby should be incorporated and entire document proof-read by a native English speaker prior to indexing of this write-up.

**Response: **All suggestions have been accepted and incorporated.
